# Hospital and Institutional News

**Published:** 1912-01-27

**Authors:** 


### HOSPITAL AND INSTITUTIONAL NEWS.

### A HOSPITAL IN REVOLT.

A curious spirit of antagonism to the King Edward VII. Hospital Fund Committee has manifested itself in certain quarters lately. The rupture between the Fund and the managers of St. George's Hospital will be fresh in everyone's recollection; and now a similar spirit of discontent has sprung up at the Evelina Hospital. The chairman of the Committee of Management of this institution, Mr. D. Malcolm Scott, has sent to the daily Press a letter setting forth the position which he and his committee have thought fit to take up. Briefly, the King's Fund three years ago recommended to the Evelina Hospital the appointment of an inquiry officer, and contributed £150 to the cost, which was rather more than £200 annually. Having thus submitted to the authority of the King Edward Fund, the Evelina managers next decided to start a Samaritan Fund, out of which the inquiry officer might relieve the necessities of deserving patients. Then in 1910 a legacy of £3,620 was received by the hospital, and it was resolved under legal advice to transfer this legacy to the Samaritan Fund. Mr. Scott argues that the legacy was left unconditionally and therefore that his committee is justified in allocating it as may be thought desirable. The Committee of the King's Fund takes the opposite view, and requires that the money be paid to the general fund. Failing such restitution, the Evelina Hospital was threatened with loss of its grant; and, as Mr. Scott and his colleagues refused to give way, that is what has taken place. We cannot uphold this unfortunate decision on the part of the Evelina authorities. To quarrel with the King's Fund, and to lose a substantial grant, on such a technical point is neither dignified nor businesslike. It is well known that the Committee of the King's Fund is especially watchful of the financial arrangements of the London hospitals, and that it includes the acknowledged best experts on hospital finance. The demand of the Distribution Committee was solely dictated by concern for the security of this legacy. Had the hospital agreed, not one penny of interest or principal would have been lost. While acknowledging the temperate and courteous tone of Mr. Scott's letter, we can only say that in our opinion his institution has made a serious tactical error, and is now in a false position from which it had best retreat as quickly as possible.

### AN ATTEMPT TO REVIVE HOSPITAL SUNDAY IN SHEFFIELD.

Mr. Philip K. Wake, the Chairman of the Board of Management of the Sheffield Royal Hospital, has received a letter from a generous benefactor of the Sheffield hospitals, offering to add ten per cent. to the total collections in all places of worship in Sheffield on Hospital Sunday, the 28th inst. This offer, we are glad to note, is made with a desire to increase the interest in, and the amount raised on, Hospital Sunday. That this needs doing is regrettably obvious from the fact that the amount collected on the last Hospital Sunday in Sheffield showed a decrease in twenty years of practically £400! This is hardly creditable to a prosperous city, and it is to be hoped that the above generous offer will have a stimulating effect upon the collections in the steel capital this year. It only remains for the clergy to do their duty in the old spirit, in reviving which the hospital chaplains should have the most energetic, as they certainly have the most responsible, share.

### THE NEW SECRETARY TO KING EDWARD'S HOSPITAL, WINDSOR.

Mr. Thomas Carter, who has held the appointment of Senior Assistant and Accountant in the Secretary-Superintendent's office at Addenbrooke's Hospital, Cambridge, for the past ten and a half years, has been appointed Secretary of the King Edward VII. Memorial Hospital at Windsor. Previous to taking up his duties at Addenbrooke's Hospital Mr. Carter had some years' experience with a firm of chartered accountants at Cambridge. Of his ten and a half years at Addenbrooke's, three and a half years were spent with Mr. Frank Ellet, the former superintendent, and seven years with Mr. Richard J. Coles, who succeeded Mr. Ellet, and of course is still in office. Mr. Carter is a son of the Rev. Thomas Carter, Vicar of St. Titus, Liverpool, and comes from a distinguished mathemathical family, several of his brothers being honour men of St. John's College, Cambridge. It goes without saying that the institutional staff regrets losing so well-known a member, but its congratulations and good wishes go with Mr. Carter, who leaves Addenbrooke's this week to take up his new duties at Windsor. It is worth noticing that more than one important appointment has fallen to the secretarial staff at Addenbrooke's during the past eighteen months.

### SECRETARIAL PROMOTIONS AT ADDENBROOKE'S.

Important changes and promotions have taken place in the secretarial department of Addenbrooke's Hospital, Cambridge, consequent upon Mr. Thomas Carter's promotion. Mr. H. F. J. Mann, who has been trained in the Secretary-Superintendent's office at Addenbrooke's Hospital, and who was appointed to succeed Mr. Tom B. Harrison about July 1910, when the latter was appointed Secretary to the General Infirmary, Chichester, has now been appointed to succeed Mr. Thomas Carter as Assistant to Mr. R. J. Coles, the Secretary-Superintendent.

### IMPORTANT NEW MEDICAL POST CREATED.

We understand that the London County Council has decided to appoint a deputy medical officer of health and deputy school-medical officer at a salary of £1,000 a year, and applications for the position are to be invited by public advertisement. Dr. Hamer, M.D., the new Medical Officer for the administrative county of London, recently submitted a scheme for the re-organisation of the public health department, but consideration of this matter has been postponed. The point he impressed very strongly, however, was that a deputy should be appointed, at the above salary, who would be able to assume the control of the department during the absence of the head, and with this contention the County Council apparently agrees.

### STEVEDORES AND THE LONDON HOSPITAL.

Under this heading Mr. James Anderson, the general secretary of the Amalgamated Stevedores' Labour Protection League has published in a morning and evening paper a somewhat truculent letter, in which he refuses to subscribe to an appeal on behalf of the London Hospital until the League is satisfied that patients sent by doctors who are prepared to work under the Industrial Insurance Act will be admitted. Mr. Anderson refers to what he calls a threat to refuse all patients sent by "blackleg" doctors, as he terms them. It is easy to see how such a vague charge may be used by the Ministerialist Press to excite opposition against the London and other hospitals, and therefore it may be worth while to examine the facts of the case. It could serve the hospitals no conceivable good turn for secretaries, still more exceptionally responsible public men like Mr. Morris, to utter threats of any kind in this matter. Did not the London Hospital Chairman remark the other day that the hospitals existed to give help and not to refuse it? This makes it perfectly clear that no threat of such an absurd kind could be charged against the London except under a misapprehension.

### HOSPITALS AND PREJUDICED TACTICS.

What happened, we are perfectly certain, was that a—perhaps unintentionally—garbled report was circulated of a simple statement of fact, which can be put in a nutshell. Since the main body of practitioners already has signed a declaration and has expressed its intention, in the event of the Insurance Act being forced on the country without the concession of the six cardinal points, to have nothing to do with cases sent up by doctors who have not signed the declaration, the position of the voluntary hospitals may become one of extreme difficulty. The members of their honorary staffs are also doctors, in fact are the very doctors who have signed their intention not to receive cases sent by those who have not signed. What then? Is this a prospect which any hospital secretary can look forward to with anything but misgiving: is it a prospect which any sane man whose institution depends on voluntary support would hold out as a threat? If Mr. Anderson had thought for five minutes he would never have written his letter, but that probably would not have suited the policy of certain newspapers which seem to lose no opportunity to excite animosity against our voluntary institutions.

### AN ORIGINAL HOSPITAL REPORT.

The *Leicester Daily Post* of the 18th inst. is interesting reading, because the proceedings at the quarterly court of the governors of the Leicester Infirmary included the reading of a report on the year's work and future outlook which occupies over two columns. This report of Mr. Harry Johnson proves him to be a hospital secretary of full knowledge and remarkable ability. It is, we believe, the first document of the kind, for although hospital secretaries and house governors make periodical reports, they are usually rather formal documents confined to actual figures and a statement of facts covering actual events. Mr. Johnson's report is an exhaustive summary and forecast full of interesting matter calculated to arouse the zeal of the governors and to stimulate public interest in the Leicester Infirmary, which local residents may properly regard with pride and affection. The Leicester Infirmary is very ably administered. It does most excellent medical and surgical work; its nursing staff has for more than twenty years been remarkable for the thoroughness of the training and the ability of the nurses who, in the absence of students, have had to do most of the dressings. We think it is just to say that the Leicester Infirmary is now entitled to rank with the first of British general hospitals—that is, of voluntary hospitals which are unconnected with a medical school.

### "A TRYING YEAR FOR THE MANAGEMENT."

The success of the Saturday Hospital Society is astonishing, and, in Mr. Harry Johnson's words, "is a triumph of organisation" both for Mr. Woolley, who has helped to build it up, and for the whole of the working classes in the district. The hospital's total income was £22,597, and the Saturday Hospital Society's £14,600! In legacies 1910 was much below the average. Owing to the increased price of commodities "it has been a most trying year from the point of view of the management" said Mr. Johnson, and as an instance he gave the rise in price of carbolic to 10½d. from 4¾d. per lb. The estimate for the coming year is £23,500, a sum that would have been larger did not the board charge each year with a proportion of such heavy expenses as external repairs and painting. In the spring the Children's Hospital will be reconstructed, during which process the disused nurses' home will be converted into two wards with fifteen beds each. The report closed with a really eloquent appeal for the continued support of the Leicester Infirmary and its Children's Hospital. After listening to its careful summary and judicious argument no one could doubt that Sir Edward Wood was right in referring "to the great work which Mr. Harry Johnson is doing here." Sir Edward Wood in concluding a capital speech contrasted the relatively small sum received in annual subscriptions with the splendid total derived from working-class support. To the five-guinea subscriber—an unreasonably small class—he recalled the fact that Leicester has only one infirmary and its Children's Hospital; that there were not in addition the one or two special hospitals which most towns have to support. This speech and the report are worth preserving as showing how interesting the often dreary farce of an annual meeting can be. It all depends on how much brains are put into it.

### A SUCCESSFUL COUNTY SECRETARY.

About eighteen months ago Mr. Tom B. Harrison, the assistant and stores clerk at Adenbrooke's Hospital, Cambridge, was appointed Secretary of the Chichester Infirmary. The effect of his work is evident in the result that, we understand, during the first complete year that he has been in office he had converted an adverse balance of £2,000 into a surplus of income over expenditure of £79. Mr. Harrison has introduced more than one reform and has initiated new organisations for the raising of income. His chief success has been in increasing the list of annual subscriptions. It needs no comment from us to emphasise the amount of hard work which this must have entailed.

### MENTAL HOSPITAL PATIENTS AS BUILDERS.

Last July the London County Council sanctioned an expenditure of not more than £1,000 to provide a house for the first assistant medical officer at Long Grove Mental Hospital. This sum was fixed because a similar house was actually being provided at Hanwell Mental Hospital for this very sum, and it was hoped that Long Grove could do a kindred work for the same money. With a view to keeping down expense, the foundations, drains, and water service for the house were put in by the hospital workmen with the assistance of the patients at a cost, to date, of £79 15s. The tenders which were obtained for building the superstructure varied from about £949 to £1,140. The Mental Hospital's engineer estimates that the cost of putting up the superstructure, if carried out under his supervision by labour directly employed, with the assistance of patients, would not exceed £850. It is therefore proposed to reject all the tenders received and to build the house by labour directly employed under his supervision. It is probable that this work will be as good for the patients as it promises to be from the treasurer's point of view.

### THE POSITION AT HARTSHILL

It is satisfactory to learn that the building fund for the North Staffordshire Infirmary, the recognition of the need for which followed The Hospital's Report, now amounts practically to £35,000, the amount required. On the whole, then, in spite of a total deficiency of £1,288, if legacies are not counted as current income, the New Year can be begun without undue anxiety. Colonel Heath at any rate is again president, and Mr. George Barlow has joined the general committee as its one new member.

### THE COST OF IMPROVEMENTS AT COLNEY HATCH.

The Mental Hospitals Committee of the London County Council announces that the annual estimates for the year 1911-12 for repairs to the buildings of Colney Hatch Mental Hospital will not be sufficient to meet the cost by £1,770. This is due to the fact that the following works, which it has been found necessary to carry out without delay, have been charged against the estimate although no provision for them had been made, the necessity not having been foreseen: Improvement of additional wards, £1,000; provision of verandahs at infirmaries, £240; and repairs to roof of recreation-hall, £530. It is curious to note that the Finance Committee of the Council, notwithstanding the statement of the Mental Hospitals Committee, is not at all certain that the estimates for the year even now will be exceeded, and the two committees are in communication on the point.

### "THE HOSPITAL" AND A PAPER.

Nothing better could show the low level to which average newspaper criticism has become reduced than the reception which our recent Report on the condition of the Hull Royal Infirmary has received from the local paper. In an exceedingly cautious article published on the 13th ult., our Report is extensively quoted with detailed comments of which the following may serve as an example: "If these are the worst defects that The Hospital can detect, things at the Royal Infirmary are not in such a very bad way after all." The astonishing ineptitude of this statement, the insight which it gives into the mind of the writer, whose last two words imply that the object of expert criticism is, not impartially to state the facts but to cause a sensation by calculated exposure, is, however, instructive. It shows the increasing need for responsible criticism, and the value even of a single newspaper's concern with fact. Sensationalism of blame or praise is, we fear, the food provided by most general newspapers. The technical journals, which as such are concerned with some special kind of fact, therefore have more than ever a necessary and useful function. Compare Sir Henry Burdett's Report on Hull Infirmary, which was simply a detailed recital of fact, with the comments it has created locally, and one can form a fair idea of the necessity for statements of fact to-day. This kind of fact it is the special function of a technical newspaper to provide, and the open-mouthed surprise it creates shows its tonic effect on the lay and long-suffering public.

### "INFIRMARY PATIENTS SLEEPING ON THE FLOOR."

Under this title on December 13 we drew attention to the serious condition of overcrowding which, on the account of Mr. J. R. Staddon, the medical officer, prevails in the Ipswich Workhouse Infirmary. This week we have great pleasure in publishing a letter from him, in which he says: "For the last six years I have repeatedly called the Guardians' attention to the necessity for providing more accommodation for the sick and infirm, and have been backed up by the Local Government Inspector." We may be forgiven for thinking that no Board of Guardians would require to receive more than one report of such gravity, and we are all the more ready to accept Mr. Staddon's assurance, for our experience generally has been that it is not the medical officer who is to blame on these occasions, but the committees of local Guardians, who often are deaf to everything except their own interest. Medical officers can bear witness only: they cannot enforce their recommendations; and when the power which an annual election gives to Guardians is remembered, the wonder is that medical officers do not connive at abuses more often than the most unscrupulous have been known to do. The medical officer, evidently, is not to blame for the grave conditions which he says obtain at the Ipswich Workhouse Infirmary, and our previous paragraph has done good service in making absolutely clear where the true responsibility lies.

### SERIOUS DISPUTE BETWEEN DOCTOR AND HOUSE COMMITTEE.

About a year ago the Renfrew and Clydebank Joint Hospital Board decided to adopt the Milne system for the treatment of their scarlet-fever patients in the Blawarthill Hospital. Whereupon Dr. Butchart, the medical superintendent, refused to abandon the existing method of treatment, and another doctor had to be called in. The Board then became still further enthusiastic for the Milne system, and, on the grounds of its effectiveness, decreed by resolution that for a test period no other system should be used for scarlet-fever cases. That period has now expired, and as Dr. Butchart still maintains his refusal, and as the expense of two medical men is felt to be great, the house committee last week recommended that Dr. Butchart "be relieved of his office, and a new medical superintendent be advertised for." This situation is important as it raises the whole question of the relation of house committees to the medical superintendents whom they appoint.

### A MEDICAL SUPERINTENDENT FIGHTS FOR HIS PREROGATIVE.

It will come as news to most people that such committees should usurp the medical officer's prerogative by laying down the treatment to be followed, still more so that the medical officer should be bound down to one method alone. Dr. Butchart, it seems, not merely has stuck to his guns with admirable firmness, but has, we believe, explained publicly, what every up-to-date medical officer knows, that the Milne system in the long run does not justify the claims that hasty sponsors make for it; that the system has not yet passed out of the experimental stage, and as a routine system can not possibly be said to have superseded established methods. This attitude is as reasonable as it is courageous, and Dr. Butchart is fighting for the great principle that medical superintendents in their own sphere must be given a free hand, and that no gentleman who happens to be a medical man can accept responsible positions without such freedom. We trust that, should a new medical superintendent be advertised for, every member of the profession will support this prerogative by abstaining from applying for the post, at any rate until he has made a full investigation.

### A RECORD OF PROGRESS AT RICHMOND, YORKS.

It is interesting to note the improvements that have been decided on by the committee of the Victoria Hospital, Richmond, Yorkshire, as many of them are the outcome of the suggestions made by Sir Henry Burdett, who visited the institution last September. The committee has decided apparently to do thoroughly what it has undertaken, and so the hospital has been closed for a fortnight, in order that the work may be done at one time. The operating-theatre is to have, what it has certainly needed, up-to-date hot and cold water fittings installed, and the corridor, which was undesirably chilly, is to be warmed by new radiators.

### A LADY PRESIDENT LEADS THE WAY.

Apart from certain minor improvements which, however, tend very much to guarantee smoothness of administration, such as a proper store-cupboard for the patients' clothes, a more important reform has been decided on. Thanks to the lively sense of responsibility which the president, the Marchioness of Zetland, feels towards her hospital, and to the deep impression which Sir Henry Burdett's remarks upon the condition of the mortuary made upon her, it has been decided to have its walls white-tiled throughout. Her Ladyship indeed has set an example by promising to give candlesticks and a table for the complete furnishing of the building. This progressive attitude reflects the greatest possible credit upon the hospital's committee, and is a useful example of the good which can result from an interchange of views between a committee of management and an independent authority, who has no other object but to record his impressions of their institution in the light of a lifetime's experience of hospital work.

### TURNING THE TABLES BY A HOUSE COMMITTEE.

The overcrowding at Worcester Workhouse Infirmary recently led the house committee to draw up a scheme of reform. Their scheme was rejected, whereupon the house committee, apparently unwilling to be outdone, decided that the objectors to their scheme should have the responsibility of dealing with the matter. The special committee thus formed recommended an alteration, the conversion of the nursery into a relief ward, which has now been adopted. This turning of the tables seems to have satisfied both parties.

### THE MANSFIELD HOSPITAL EXTENSION SCHEME.

In June last we had the pleasure of recording the efforts that were being made by Mr. H. E. Hollins, as chairman of the Mansfield Hospital, Nottingham, towards raising the £10,000 which was required to build the two isolation wards and the better accommodation for the domestic staff which had been decided on. The hospital's patron, the Duke of Portland, who gave £1,000 six months ago, visited the hospital last week and most generously has doubled his present. The Board, therefore, is now in the position of having raised some £5,000, half, that is, of the total required sum. This enables the Board to act on its decision to start building as soon as half the necessary amount was in hand. It is only fair to add that the colliery owners and their workers apparently have responded to Mr. Hollins' appeal in a way that is as surprising as it is gratifying, for the industrial development of Mansfield has been faster than its recognition of hospital needs. On the present occasion, however, whether due to the Duke's example or not, the locality has responded creditably to the institution's claims, and we hope Mr. A. H. Limb, the Secretary, will let us know as soon as the architect has been decided on and the work begun. It should be added that the extension scheme was projected as a memorial to King Edward.

### AN ENTERPRISING COTTAGE HOSPITAL.

Mr. R. R. Redmayne, honorary secretary and treasurer of the Lichfield Nursing Home and Cottage Hospital, and the chairman of the executive committee, Mr. H. M. Morgan, are to be congratulated on the local support which their institution is consolidating. No sooner, apparently, is a deficiency of £87 announced than up jumps a subscriber with a cheque for £90. More than this, a far-seeing believer in annual subscriptions, who prefers to remain anonymous, has promised £10 a year if nine others can be found to give an equal amount. Once again, the City Council has given the balance (£28) of the Queen Victoria Memorial Fund to the building fund. Mr. Redmayne should feel in clover; and perhaps as an outward sign of increasing importance, the office of president has been created, and its first holder is Sir Richard P. Cooper, Bart., while Prebendary Bolton has been re-elected chairman of the general committee. We were under the impression that on its growth to an institution of fourteen beds the office of president previously had come into existence and that indeed the Rev. C. N. Bolton had been its holder. Mr. Redmayne no doubt could explain how this arose, as also why the old style of nursing home is still adopted. The stage this represents has, surely, been outgrown.

### A LOSS TO THE CHESTER INFIRMARY.

By the death of Mr. John Taylor, F.R.C.S., J.P., the Chester Infirmary has lost one who in the words of the Chairman of the Board, Colonel Evans-Lloyd, has been for 46 years "the backbone" of the infirmary. Mr. Taylor for two years was visiting surgeon, for 38 years full surgeon, and for the past six years has been on the consulting staff. A striking tribute to his personality was paid by Colonel Evans-Lloyd, who said: "Mr. Taylor was in a very special way the devoted adviser and friend" of the hospital's staff. Mr. E. Brassey warmly seconded this tribute. On July 4, 1907, a public presentation was made to him for his services to the infirmary and city of Chester.

### A MEDICAL MISSIONARY.

In view of our recent comments on the importance—indeed necessity—for a medical training for all who engage in missionary work, it is interesting to note the death of Dr. Edmund Downes at Eastbourne, after a long illness, at the age of 68. He studied at St. Mary's Hospital and qualified in 1876 at the London Colleges; he also took the M.D. of Brussels in the same year. For a few years he held a commission in the Royal Artillery, but then directed his efforts to the missionary field. Dr. Downes joined the Church Missionary Society, and for several years did enthusiastic work for their mission in Kashmir. His example was one to be followed. He contributed several papers on surgical subjects, and became in 1884 a Fellow of the Royal College of Surgeons of Edinburgh. After leaving the Church Missionary Society Dr. Downes settled in Eastbourne, where he practised for many years, but he had not been in active practice for some time past. He was a member of the Harveian Society. The medical training of missionaries was, we believe, very dear to his heart.

### A WOMAN'S INDIAN MEDICAL SERVICE.

It is generally true to say that all Indian questions are expert questions, and that on the great majority of Indian questions the Englishman is a layman. Nevertheless, the proposal for a Women's Indian Medical Service seems to have much to recommend it on the grounds of common sense. We are told by correspondents who claim to have acquired their knowledge on the spot that the mortality among Indian mothers, and Indian women generally, is inordinately high because custom and caste preclude them from being attended by a male practitioner, and, further, such native women doctors as are available are said to be ignorant in the extreme. To combat this terrible evil, this needless waste of life, Lady Dufferin's Fund, and that started by Lady Curzon have been the chief agencies, but their prospects are not good enough to attract the best women practitioners, and their chief value has been to show the feasibility and urgency of the work. The possibility of Indian hospitals staffed by women doctors has been proved, and the results, there seems no doubt, justify extension. To be adequate, however, such extension must be placed in Government hands, for that is the sole agency capable of grappling with the colossal proportions of a scheme that would be at all adequate to the population. Any women's Indian medical service, however, would have to provide for the medical training of native women, who, we are led to believe, would be prepared, to some extent at any rate, to use any opportunities afforded them. We believe that the inauguration of such a service, coupled, as has been suggested, with the name of the Queen-Empress, would do more for India than any other one measure, and that the service very quickly would repay its initially great cost. If Dr. Emma Slater, whose proposals we noted in December last, is right, the Government of Bombay set an example long ago that should prove now doubly instructive.

### TWO NEW MEDICAL PAPERS.

The last two months have witnessed the birth of two new arrivals in the fairly crowded world of medical journalism. In December of last year the first issue of a new quarterly was published by the management of the Sleeping Sickness Bureau. This new manifestation of the energies of the committee and of its capable director, Dr. A. G. Bagshawe, is named the *Kala-Azar Bulletin*. It resembles in general format the well-known *Sleeping Sickness Bulletin*, and is under the sub-editorship of Dr. C. M. Wenyon, of the London School of Tropical Medicine. As a general rule, no charge will be made for copies of this publication.

### AN UNANNOUNCED REVIEW.

The other new arrival has been sprung somewhat suddenly upon the profession. Without any of the usual "puffs preliminary" it has quietly appeared, under the style and title of the *Universal Medical Record*. The first number is that for January, and subsequent issues will appear on the fifteenth of each month. The manner and matter of this new monthly periodical are alike excellent. Illustrations apparently form no part of the scheme so far. As for matter, the new venture may perhaps be described as a compromise between the *Practitioner* and the *Medical Review*, two admirable papers whose orbits lie fairly wide apart. It is not a blend of these two; it has a distinct individuality of its own, but is also slightly reminiscent of the monthlies just mentioned. In this first number the premier place is given to one of those gem-like essays by Sir T. Clifford Allbutt, of which he seems to be an inexhaustible mine, but whose lustre is largely due to the industrious polishing of their distinguished author. The ubiquitous Mr. Moynihan comes, inevitably, next; there can be few medical papers to which this voluminous but always arresting author has not contributed. The whole makes up a most attractive number, and one which is highly to the credit of Mr. G. S. Elliston, an old Guy's man, who is editing the new venture.

### UNJUSTIFIABLE OPERATIONS.

The recent report of an action against an American hospital surgeon for "performing an unjustifiable operation" recalls the case of the Berlin physician which some years ago created a mild sensation in the German capital. This gentleman was one of the assistants of the late Professor von Leyden at the Charité, and conducted what we believe was the first operation for resection of the lung on the human subject. The patient was a young girl who agreed to the operation, which was carried out after the *modus operandi* had been tried and perfected upon animals. The case was tubercular disease of the right apex, and the patient died two days after the operation. The relatives heard of the operation and accused the physician before the magistrate. The case came on for trial and several surgeons were called, who declared that the operation was entirely unjustified and unwarranted. The fact that the interference had been unsuccessful no doubt weighed largely with the court, although the adverse surgical criticism must also have materially influenced the judges. At any rate the physician was found guilty of manslaughter and sentenced to seven years' imprisonment. Since then the same operation has been attempted with perfect success, and it is now a recognised surgical procedure in certain cases. Gluck has extirpated nearly the whole of one lung, and abroad, where the "high-pressure cabinet" is an indispensable adjunct to every modern operating-theatre, resections of lung tissue are frequent operations. At University College Hospital a high-pressure cabinet has lately been installed and has, we believe, already been used with success. In the circumstances it seems difficult to define what constitutes an unjustifiable operation. Perhaps readers will give us their opinion on the subject.

### CHANGES AT THE GERMAN HOSPITAL.

During the last quarter the German Hospital at Dalston has been in a state of transition. The builders have been in for several months, and the whole of the operating block and the surgical wards have been rebuilt. The operating theatre has been enlarged and modernised, and the two male wards have been considerably improved in appearance. In addition private and isolation rooms have been added. The new nurses' home was opened a few months ago and is in every way an excellent, though necessarily a somewhat small, home. The hospital has recently gained two beds owing to the permanent endowments left by friends. One of these beds is endowed by the Weber family in memory of the late Lady Weber, wife of Sir Herman Weber, the senior consulting physician, who is now, we believe, the doyen of the College of Physicians in London. The hospital has lost the services of Dr. D. O'Finegan, who lately resigned his post as assistant physician to take up a country practice. His successor has not yet been elected.

### THE MEDICAL EFFECTS OF A MILD WINTER.

The old saw about the "green winter" and the "full kirkyard" was under discussion in the lay press recently, owing to the unusually large number of octogenarians and nonagenarians whose names were appearing in the first columns of the newspapers. Whether or not a mild winter really does increase the mortality among the aged, the Registrar's returns for December are evidence that it agrees well with those at the other extremity of life. Thus in the four weeks from December 3 to December 30 there were in London but 50 deaths from measles, against a decennial average of 151; 37 from whooping cough, as against 68. The figures relating to notifications tell much the same tale, though not quite to so marked a degree. Only 69 cases of enteric fever, against an average of 152, is quite good; 903 of scarlet fever, against 1,312, is also satisfactory, as is the total absence of small-pox compared with 69 cases a year on the average for 1901-10. Diphtheria, unfortunately, has been notified 683 times, as against an average of 622. But on the whole it is clear that the mild weather prevailing up to the end of last year has favoured the chances of the children, even if it has prejudiced those of the veterans.

### THIS WEEK'S DRUG MARKET.

Business in the drug market has been quieter this week, and the number of price changes unusually small. Several articles, including balsam of tolu, cream of tartar, and saffron, have a tendency towards slightly lower values, but there are practically no changes in price in an upward direction. The position of opium is well maintained.

